# Assessing dioxin emissions change in the transition from landfilling of MSW to waste-to-energy

**DOI:** 10.1007/s11356-025-37006-x

**Published:** 2025-10-11

**Authors:** Amirsohrab Falsafi, Amirmehrab Falsafi, Mariam Abdulkareem, Mika Horttanainen

**Affiliations:** Department of Sustainability Science, Lappeenranta-Lahti University of Technology LUT, Yliopistonkatu 34, 53851 Lappeenranta, Finland

**Keywords:** Dioxin emissions, Landfill fire, Waste-to-energy, Municipal solid waste, Waste incineration

## Abstract

The term “dioxins” encompasses a group of chlorinated organic pollutants, including polychlorinated dibenzo-dioxins (PCDDs) and polychlorinated dibenzofurans (PCDFs), which impact human health, affecting immunological, neurological, endocrine, and reproductive systems. Activities like burning fuels, waste incineration, landfill fires, and certain industrial processes are major sources of dioxin emissions. There has been much debate on the quantity of dioxins released by waste incinerators but considerably less debate has been carried out on the dioxin emissions of landfill fires. Thus, this study evaluates dioxin emissions changes in countries that have transitioned from landfill to waste-to-energy (WTE) in a specific period. This study analyzes data from selected countries, including the UK, Austria, Poland, Ireland, Norway, Lithuania, Finland, and China, and applies parametric estimation methods to determine changes in dioxin emissions during the transition from landfill to WTE. The results of this study indicate that reducing landfilling leads to a significant decrease in dioxin emissions. In the studied countries, dioxin emissions decreased in the range of 27% and 94% per mass unit of waste treated by WTE and landfill methods. This reduction emphasizes the potential of WTE to increase environmental sustainability compared to traditional landfilling. Also, it confirms that the reduction of landfill fires is the main factor in the reduction of dioxin emissions. The study’s insights may serve as guidance for underdeveloped and developing regions seeking to mitigate dioxins and other pollution through modernized waste treatment infrastructure.

**Supplementary Information:**

The online version contains supplementary material available at 10.1007/s11356-025-37006-x.

## Introduction

Disposing of municipal solid waste (MSW) has become a critical and contentious challenge for local governments worldwide. Numerous challenges in managing MSW arise from the increasing waste generation driven by population growth in developing and developed countries (Cherubini et al. 2009). MSW can be managed through options such as landfilling and incineration among others. While landfilling is a cost-effective way for disposing of waste (Dąbrowska et al. 2018; Šourková et al. 2020), unregulated usage of landfills may lead to landfill fires, which can have adverse effects on the environment (Zhang et al. 2017; Cudjoe et al. 2020; Cudjoe and Acquah 2021). Waste incineration on the other hand can decrease waste by as much as 70% (Luo et al. 2019; Wang et al. 2017), but also has significant environmental consequences such as the release of dioxins into the environment, same as for landfill fires. Dioxins are polychlorinated dibenzofurans (PCDFs) and polychlorinated dibenzodioxins (PCDDs), emitted through a wide range of burning processes, including waste incineration and landfill fires (Kulkarni et al. 2008; Ruokojärvi et al. 1995). Since dioxins are among the most harmful substances released into the environment by combustion, there has been much debate on the quantity of dioxins released by waste incinerators but considerably less debate has been carried out on the dioxin emissions of landfill fires (Schuhmacher and Domingo 2006; Ruokojärvi et al. 1995).

Dioxin emissions from waste management practices have been widely studied, with a strong emphasis on controlled combustion processes like incineration. However, landfill fires remain an underrepresented source of dioxin pollution, particularly in developing regions. Recent studies have indicated significant emissions from open burning and landfill fire incidents, particularly in India and China (Ajay et al., 2021; Zhang and Li, 2017). Additionally, the UNEP toolkit has compiled multiple global studies on dioxin emissions and established default emission factors applicable worldwide. The inclusion of these resources strengthens the accuracy of emission assessments and ensures alignment with the latest methodologies.

Furthermore, Weichenthal et al. (2015) documented the impact of a landfill fire on ambient air quality in Iqaluit, Canada, reinforcing concerns about the widespread consequences of such events. These findings underscore the importance of transitioning away from uncontrolled waste disposal methods to technologically advanced WTE solutions, especially in regions with high waste generation rates.

Furthermore, the monitoring of Persistent Organic Pollutants (POPs) plays a crucial role in assessing their environmental and human health impacts. The *Global Monitoring Plan on Persistent Organic Pollutants*, coordinated by the United Nations Environment Programme (UNEP) and supported by Örebro University’s MTM Research Centre, aims to generate high-quality data on POPs in core environmental and biological matrices worldwide (Örebro University 2022). These efforts, including interlaboratory assessments and capacity-building initiatives, enhance the accuracy of global dioxin emission evaluations and contribute to the implementation of the Stockholm Convention.

According to the World Health Organization (WHO, 2016), people exposed to high levels of dioxins for a short period of time are at risk of developing dermatitis and liver dysfunction. A long-term exposure to these chemicals can result in an impairment of the immune system, neurological system, endocrine system, and reproductive system. As a result, incinerators are now required to conduct more rigorous monitoring of dioxin emissions at start-up and shut-down periods, to help reduce the release of persistent organic pollutants (POPs). Also, through waste-to-energy (WTE) technologies, dioxin emissions have significantly reduced throughout Europe by efficiently managing municipal waste that would otherwise end up in landfills, and potentially resulting in landfill fires. As an example, the Swedish WTE industry has been reported to be associated with significant reductions in dioxin emissions, down from 100 g in 1985 to less than 1 g in 2015, even as the volume of waste treated thermally has more than doubled (CEWEP 2022).

Several estimation methods exist for assessing open-fire waste burning emissions. The IPCC protocol (IPCC 2006) provides methodologies for estimating emissions from incineration and open burning based on factors such as waste composition and combustion efficiency. Additionally, NASA’s Fire Information for Resource Management System (FIRMS) employs satellite-based thermal anomaly detection to monitor open fires globally. However, these methods have limitations when applied specifically to landfill fires, as they generalize emissions without distinguishing between controlled open burning and spontaneous landfill fires, leading to potential inaccuracies in dioxins estimates (Zheng et al., 2021). Remote sensing techniques can detect fire events but may struggle to differentiate landfill fires from other combustion sources. Furthermore, landfill fire emissions vary based on waste composition, combustion conditions, and fire frequency, factors that are often underrepresented in generalized models.

However, it is important to note that precise statistics for dioxin emissions from landfills in Europe are not readily available, indicating a potential area for further research and regulation. Dioxin pollution is being monitored and regulated more effectively within the EU, indicating a proactive approach to managing and minimizing environmental and public health risks (Zero Waste Europe 2023). The European suppliers of waste to energy ESWET report (ESWET, 2022) emphasize that well-managed WTE facilities in the EU emit extremely low concentrations of dioxins, often below the limit of detection, thanks to advanced combustion control and pollution abatement systems. This supports the claim that WTE can significantly reduce dioxin emissions compared to traditional landfills. Furthermore, in the report, dioxin emissions from landfill fires are also mentioned to contribute significantly with an emission factor for surface landfill fires to be 5000 times greater than the emission factor for incineration of MSW.

Several in-depth studies have been conducted on various sources of dioxin emissions, including waste combustion sources, chemical-industrial sources, and other thermal sources. To minimize the harmful effects of dioxin emissions, the mass of dioxins should be controlled during municipal solid waste incineration (MSWI). According to Dwyer and Themelis (2015) research, “dioxins” can be classified into two categories based on their sources: controlled industrial sources and open burning sources including landfill fires. Based on an assessment by Dwyer and Themelis (2015), the amount of dioxin emissions in the United States was estimated in 1987 and 2012. As a result of controllable sources, the total emissions from controlled sources decreased by 95.5% in 2012, from 14 kg Toxic Equivalent (TEQ) in 1987 to 0.6 kg TEQ in 2012. Moreover, open-burning sources have increased their emissions from 2.3 kg TEQ in 1987 to 3 kg TEQ in 2012. Additionally, the release of dioxins in the U.S. has reduced from 14 000 g in 1987 to 1 400 g in 2000 as a result of improved waste management and combustion practices (Dwyer and Themelis 2015). Despite this, sources such as landfill fires are a significant source of dioxin emissions, contributing significantly to the overall environmental load of these toxic chemicals, and continue to pose challenges due to their unpredictable nature and the difficulty in controlling such emissions (Environmental Protection Agency, 2019).

Wei et al. (2022) reported that China does not have a current regional inventory of dioxin emissions from MSWI, even though MSWI has grown rapidly in recent decades. The dioxin emissions inventory was compiled from information collected from government and corporate websites in mainland China, leading to MSWI emitting 22.56 g-TEQ of dioxins in 2020.

Another study, conducted by Viel et al. (2011), investigated the nature of dioxins soil contamination surrounding the Besançon incinerator in France, to determine whether dioxins could be attributed to more than one source of emission. Because of the sampling site selection and the high similarity in congener profiles, as well as the absence of other polluting industries in the study area, it was concluded that the presence of dioxins was caused solely by the MSWI. The study area is expected to see a gradual decline in dioxins concentrations in soil after the closure of the most polluting furnaces and the replacement of the furnace with an advanced Air Pollution Control system. Also, there have also been several studies indicating that, by switching from landfilling to waste-to-energy, GHG emissions and harmful pollutants like dioxins are reduced (Luckow et al., 2010; Tan et al. 2015; Wang et al. 2017).

Based on the significant amounts of dioxin emissions from both WTE technology and landfill fires, this study examines how dioxin emissions are changing as waste management practices transition from landfilling to WTE and aims to assess the changes in dioxin emissions in countries that have made a significant transition from landfill to WTE treatment, utilizing parametric estimation to determine these changes. Also, it is noteworthy to mention that the research presented in this article is an extension of the concepts and methodologies initially explored in the master’s thesis by A.S. Falsafi (2023).

## Materials and methods

The selection of case countries was based on a quantitative assessment of changes in waste management practices, specifically focusing on countries that exhibited significant reductions in landfill use alongside substantial increases in WTE adoption. An initial evaluation was conducted for all countries based on available waste treatment data. Countries included in this study demonstrated a landfill reduction between 5 and 50% and a corresponding WTE increase between 8 and 35% during a specific period. These trends were identified using Eurostat (2022) and the China Statistical Yearbook (2020), ensuring that the selected countries had undergone a meaningful transition from landfill-based disposal to WTE solutions. Following this evaluation, eight countries were considered: the United Kingdom (UK), Austria, Poland, Ireland, Norway, Finland, Lithuania, and China. The transition periods for each country are presented in Table 1. Based on the data from these countries, we applied parametric estimation to assess dioxin emissions from incinerators and landfill fires, as further discussed in sections.

**Table 1:** Input data

Countries	Transition period	F_I_ (μg I-TEQ/t_I)_^a^	WTE (kt)^b,c^		Landfill (kt)^b,c^	
			Start point	End point	Start point	End point
**Ireland**	2008 to 2014	0.0525	82	893	1939	537
**Lithuania**	2013 to 2020	0.0525	92	349	798	220
**Austria**	2005 to 2020	0.0440	1310	2652	535	137
**Poland**	2012 to 2020	0.0525	51	2823	8085	5218
**Finland**	2010 to 2019	0.32	556	1908	1136	30
**Norway**	2006 to 2013	0.0440	675	1446	390	52
**UK**	2005 to 2018	0.17	2942	12,615	22,569	4613
**China**	2010 to 2017	0.17	23,000	102,000	95,000	120,000

^a^Wei et al. (2022)

^b^Eurostat (2022)

^c^China Statistical Yearbook (2020)

It should be noted that industrial point sources such as metal smelting and coal-fired power generation are acknowledged contributors to national dioxin inventories, but they lie outside the scope of this work, which compares only the waste-sector alternatives of landfilling (with its attendant fire risk) and controlled waste-to-energy incineration.

### Dioxin emissions from incinerator

Based on the article by Cudjoe and Acquah (2021), Eq. (1) is derived to calculate the amount of dioxin emissions from the burning of waste in incineration plants. There are two factors that must be considered in this calculation. One is the amount of MSW that is incinerated, and the other is the value of the dioxin emissions factor. Dioxin emissions factors for incinerators in specific countries are detailed in the study by Wei et al. (2022); however, data for some countries were missing. In the case countries, it was assumed that the dioxin emissions factor from a country with a comparable volume of waste that is processed through WTE processes would apply. As an example, the dioxin emissions factor for Ireland and Lithuania was that of Poland, while the factor for Norway was that of Austria. In the case of the UK, the assumed emission factor for dioxins was the same as that of China. In Table 1, dioxin emissions factors are summarized.

1 $$DI={F}_{I}\times {M}_{WTE}$$

where

*DI*: annual dioxin emissions for waste incineration (mg-TEQ)
*F*_*I*_: dioxin emissions factor (μg-TEQ/t _MSW_)
*M*_*WTE*_: total mass of WTE (kt) in treatment of MSW per year

### Dioxin emissions from landfill fires

Estimating dioxin emissions from landfill fires remains challenging due to the limited availability of country-specific data on their frequency, intensity, and associated emissions. Research data on dioxin emissions from landfill fires is particularly scarce, necessitating the development of a rough calculation method to approximate emission magnitudes for different countries. While methodologies such as the IPCC protocol (IPCC 2006) and remote sensing tools like NASA’s FIRMS provide insights into open-burning emissions, these approaches do not fully capture the distinct characteristics of landfill fires, which differ from controlled open burning. To address these limitations, a parametric estimation approach was developed, leveraging country-level waste treatment data to refine emission assessments. This method integrates assumptions from existing studies while compensating for the absence of direct measurements. The estimation framework applied in this study is adapted from Dwyer and Themelis (2015) and incorporates key parameters such as estimated landfill fire occurrences, waste disposal trends, and combustion characteristics. Additionally, the concept of Eq. (2) is derived from this methodology to estimate the amount of dioxins released from landfill fires.

2 $$DL=NLF\times WBL\times {F}_{LF}$$

where

*DL*: total annual amount of dioxin emissions (mg-TEQ/a) from landfill fires.

*NLF*: number of landfill fires in each year.
*WBL*: the mass of waste burnt per landfill fires (kt).
*F*_*LF*_: dioxin emissions factor from landfill fires (ng TEQ/kg _burned_).

Dioxin emissions can be estimated using Eq. (2), which requires the dioxin emissions factor, the number of landfill fires, and the amount of waste burned. It should be noted that the available data for calculating these emissions were limited to the United States in 2011 and Finland in 1992. The U.S. data from 2011 was used since it is more recent than the Finnish data. Moreover, these data are assumed to be uniform across all case countries.

Because of a lack of data in the concerned countries, Eq. (3) was developed to calculate the ratio of MSW incinerated in landfills to the total amount of waste deposited. The ratio is then applied to other countries that do not have specific data. Additionally, to determine the amount of MSW burned annually in landfill fires (*MLB*), one must multiply the number of landfill fires by the amount of MSW that is combusted in each incident. ${M}_{LF}$ symbolizes the total amount of MSW incinerated during these fires, relative to the annual mass of waste managed by landfills and it is used to get the approximate amount of MSW burned in landfill fires in each country per year. There is a wide variation in the probability of landfill fires between countries as a result of climatic conditions, how MSW is managed, and how much methane is captured. Despite this, no superior data have been documented or analyzed in this context. Thus, this generalization is used to estimate the potential scale of dioxin emissions from landfill fires. The data related to landfill fires and the amount of waste burned are derived from Dwyer and Themelis (2015), while the amount of waste directed to landfills is based on data from the United States Environmental Protection Agency (2013).

3 $$M_{LF}=\frac{MLB}{M_L}=\frac{11000\;fires/year\times225\;t/fire}{131\;770\;000\;t/year}=\mathrm{0,0187}$$

where

*M *_*LF*_: the ratio of amount of waste burned in landfill to the total amount of MSW treated by land fill in US per year which is constant number 0.0187 (kt _burned_/kt _landfill_).
*M*_*LB*_: total mass of waste burned in landfill fires in a year (multiplication of number of fires occurs per year by the amount the mass of waste burned in landfill fire in each fire), t/year.
*M*_*L*_: total mass of landfilling (kt) in treatment of MSW per year.

Available national data on waste actually consumed in landfill fires are extremely limited. The best-documented cases are the United States in 2011 (≈ 11,000 fires year⁻^1^, 225 t fire⁻^1^; 1.9% of land-filled MSW; Dwyer and Themelis 2015) and Finland in 1990–1992 (380 fires year⁻^1^, 84,000 t year⁻^1^; 2.9%; Ettala et al. 1996). We therefore apply their mid-point, 1.87%, as a representative global value. Varying this share from 0.5 to 5% altered the calculated percentage reduction in dioxin intensity by ≤ 8 percentage points and did not affect country rankings. Absolute totals should thus be viewed as order-of-magnitude, and routine measurement of waste lost in landfill fires remains a priority.

Furthermore, regarding the obtained ratio from Eq. (3), ${M}_{LF}$ (0,0187) as shown in Eq. (3) is used for all case countries. This factor means that about 2% of all the landfilled waste is estimated to be burned in landfills. Also, the dioxin emissions factor of landfill fires which is taken from article of Dwyer and Themelis (2015) is assumed to be constant number for all selected countries with a value of 700 ng TEQ/kg _LF_. Finally, by multiplication of these two constant numbers, Eq. (4) is simplified with one constant number. Then, we multiply this number for each selected country by the amount of landfilled waste to have a very approximate estimate of the amount of dioxin emissions in landfill fires Dwyer and Themelis (2015).

4 $$DL=\mathrm{13,09}\frac{mg}{k{t}_{landfill}}\times {M}_{L}$$

where

*DL*: total amount of dioxin emissions (mg-TEQ/a) from landfill fires.

*13,09*: the constant number $(\frac{mg}{{kt}_{L}})$ for each selected countries which is result of multiplication of dioxin emissions factor of landfill fires by *M *_*LF*_ (700 ng TEQ/kg _burned waste_ * 0.0187).

*M *_*L*_: total mass of landfilling (kt) in treatment of MSW per year.

Although the constant emission factor of 0.7 µg TEQ/kg (700 µg/t) adopted from Dwyer and Themelis (2015) provides a pragmatic first approximation, published landfill-fire factors span at least two orders of magnitude. Field-scale studies report values as low as 30 µg TEQ/t for damp, compacted dumps (UNEP Toolkit, 2023 update) and up to 3000 µg TEQ/t for loosely arranged, oxygen-limited burns (UNEP default for uncontrolled waste combustion). A recent fire at the Brahmapuram dump yard in India yielded 167 µg TEQ/t (CSIR-NIIST 2021). We therefore performed a sensitivity analysis using EF = 200 µg/t (lower bound) and EF = 3 000 µg/t (upper bound). The relative dioxin-reduction achieved by transitioning from landfill to WTE changed by less than ± 6 percentage points in every case, confirming that the study’s qualitative conclusions are not contingent on the chosen factor. Nevertheless, country-specific EF measurements remain a priority for improving absolute‐value accuracy.

Furthermore, Eq. (5) provides a formula for calculating the amount of dioxins released by landfills and WTE facilities annually per kiloton of MSW processed. As a result of this calculation, it can be analyzed dioxin emissions trends over a transition period, illustrating the impact of increasing WTE use and reducing landfill reliance on dioxin emissions during this period.

5 $$D=\frac{DL+DI}{{M}_{LI}}$$

where

*D*: amount of dioxin emissions per kt of landfilled and incinerated waste (mg-TEQ/kt _LI_).
*DL*: total amount of dioxin emissions from landfill fires (mg-TEQ/a).
*DI*: total amount of dioxin emissions from WTE plants (mg-TEQ/a)
*M *_*LI*_: sum of landfilled and incinerated waste (kt/a).

In the final step, Eq. (7) is created to show the annual changes of dioxin emissions from landfill fires and WTE based on per kt of MSW treated by landfill and WTE during the transition period to show which countries have had the most significant reduction. Then, Eq. (8) shows percentage of increasing or decreasing rate per kt of MSW treated by landfills and WTE during the transition period.

6 $$\Delta D={D}_{2}-{D}_{1}$$

7 $$AD'=\frac{D_2-D_1}{T_2-T_1}$$

8 $$\Delta D =\frac{{D}_{2}-{D}_{1}}{{D}_{1}}\times 100$$

ΔD${^{\prime}}$ : changes in the amount of dioxin emissions from landfill fires and WTE during transition period per year (mg-TEQ/(kt_LI_ a)).
*ΔD*: changes in the amount of dioxin emissions from landfill fires and WTE during transition period (mg-TEQ/(kt_LI_ a)).
*ΔD*: the percentage of dioxin emissions changes rate per kt of MSW treated by landfills and WTE during the transition period (*%).*
*D*_*1*_: total amount of dioxin emissions from landfill fires and WTE at the starting year of transition period (mg-TEQ/(kt_LI_ a)).
*D*_*2*_: total amount of dioxin emissions from landfill fires and WTE at the end year of transition period (mg-TEQ/(kt_LI_ a)).

T_1_: starting year of transition period in each case countries.
T_2_: ending year of transition period in each case countries.

## Results

Table 2 displays the estimated total dioxin emissions resulting from landfill fires and WTE facilities across case countries at the beginning and end of the transition period. According to the results, there is a considerable reduction in dioxin emissions in all the examined countries, associated with increased use of WTE technologies and a decrease in landfilling during the transition period. Despite this, China demonstrates a contrary trend, experiencing a slight rise in dioxin emissions. The main reason for this is the rapid increase in MSW generation in China and a persistent reliance on landfills despite a considerable expansion in WTE adoption. Specifically, the volume of waste landfilled in China was 96 Mt at the start of the transition period in 2010, increasing to 120 Mt by 2017. Concurrently, the use of WTE in China increased by 270%-point over the same period, with the amount of waste processed by WTE technologies rising from 23 to 85 Mt (China Statistical Yearbook, 2020). To facilitate a more comprehensive and accurate analysis, as well as to enable comparisons with pioneering countries in this transition, formulas were developed to estimate potential dioxin emissions per kt of waste processed using WTE and landfill methods.

**Table 2:** Total mass of dioxin emissions from WTE and landfill fire

Countries	Transition period	*DI* + *DL* (g-TEQ)	*DI* + *DL* (g-TEQ)
		Start point	End point
**Ireland**	2008 to 2014	25	7.1
**UK**	2005 to 2018	300	61
**Norway**	2006 to 2013	5.1	0.74
**Finland**	2010 to 2019	15	0.95
**Poland**	2012 to 2020	106	68
**Austria**	2005 to 2020	7.1	1.9
**Lithuania**	2013 to 2020	10	2.9
**China**	2010 to 2017	1300	1600

To further enhance comparability, the amount of dioxin emissions per year has been calculated separately for each country from both WTE and landfill scenarios. These calculations provide a clearer understanding of the contributions of different MSW management practices to overall emissions. While the primary focus of this study is to analyze the transition from landfill to WTE and compare emissions reductions across countries, individual inventories for each waste management approach have been included in the Supplementary Material to offer additional detail for readers interested in specific national emission values.

**Fig. 1:**
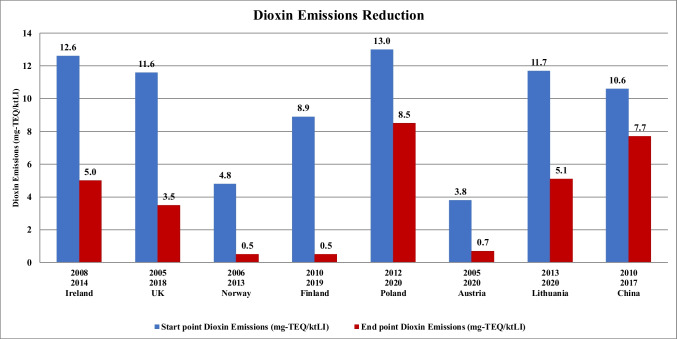
Dioxin emissions reduction in selected countries during the transition from landfilling to WTE

To better highlight these trends, Fig. 1 visually compares the reduction in dioxin emissions per kt of MSW treated by WTE and landfilling across all studied countries. The figure highlights that Ireland, the UK, Finland, and Lithuania achieved the most significant reductions, with dioxin emissions decreasing by more than 50%. On the other hand, Austria and China show the least reduction, reinforcing the earlier observation that China’s high waste generation rates and continued landfill reliance hinder its emission reduction potential.

Table 3 and Fig. 1 together provide valuable insights, facilitating comparisons among countries and identifying reasons for greater reductions. Based on this analysis, it is possible to gain a thorough understanding of the factors contributing to dioxin emissions reductions in different waste treatment methods. Also, as shown in Table 3, Finland, Ireland, Lithuania, and the UK have experienced the highest reductions in dioxin emissions per kt of MSW treated by landfill and WTE methods. Notably, Ireland achieved these reductions in the shortest timeframe and saw the most substantial annual decrease, as indicated by *ΔD*’ in Table 3. Specifically, Ireland managed to reduce emissions by 1.3 mg-TEQ/(kt_LI_ a).

**Table 3:** Changes in dioxin emissions in each country

Countries	Transition period	*D*_1_(mg-TEQ/kt_LI_)	*D*_2_(mg-TEQ/kt_LI_)	*ΔD* (mg-TEQ/kt_LI_)	*ΔD'* (mg-TEQ/(kt_LI_ a)	*ΔD* (%-point)
Ireland	2008 to 2014	12.6	5	− 7.6	− 1.3	− 61
UK	2005 to 2018	11.6	3.5	− 8,1	− 0.6	− 69
Norway	2006 to 2013	4.8	0.5	− 4.3	− 0.6	− 90
Finland	2010 to 2019	8.9	0.5	− 8.4	− 0.7	− 94
Poland	2012 to 2020	13.0	8.5	− 4.5	− 0.5	− 35
Austria	2005 to 2020	3.8	0.7	− 3.1	− 0.2	− 82
Lithuania	2013 to 2020	11.7	5.1	− 6.6	− 0.9	− 57
China	2010 to 2017	10.6	7.7	− 2.8	− 0.41	− 27

It was found that Ireland drastically reduced its reliance on landfilling during the transition period. In 2008, 60% of its waste was landfilled, which decreased to 20% by the end of the period. Conversely, the WTE industry saw substantial growth, with the share of waste treated by WTE methods increasing from 3% in 2008 to 35% in 2014. This transition is evident in Fig. 1, where Ireland shows one of the largest drops in dioxin emissions.

Moreover, the EU’s Landfill Directive is influencing policies by setting ambitious targets to reduce landfill usage. By 2035, the directive aims to limit the share of municipal waste landfilled to just 10%, encouraging member states to prioritize waste prevention, reuse, recycling, and recovery. This policy has a profound impact on EU countries’ waste management strategies (European Commission, 2023).

This study estimates that Finland and Lithuania have achieved significant annual reductions in dioxin emissions, with reductions of 0.7 mg-TEQ/(kt_LI_ a) and 0.9 mg-TEQ/(kt_LI_ a) respectively. Other countries, including Norway, the UK, and Poland, have also seen commendable reductions, ranging from 0.5 to 0.6 mg-TEQ/(kt_LI_ a). However, Austria and China have experienced the smallest changes among all case countries, with reductions of 0.2 mg-TEQ/(kt_LI_ a) and 0.4 mg-TEQ/(kt_LI_ a), respectively.

The analysis reveals that Austria, which has focused extensively on recycling, maintains low dioxin emissions. According to data presented in Table 3, dioxin emissions in Austria at the beginning of the transition period were 3.8 mg-TEQ/kt_LI_, decreasing to 0.7 mg-TEQ/kt_LI_ by the end. In contrast, the lesser reduction in China is linked to higher emission rates from MSW incineration, and the huge amount of MSW is going to the landfills that lead to an increase the risk of landfill fires and dioxin emissions. This discrepancy, as identified by Cheng and Hu (2010), stems from differences in incineration technologies and flue gas cleaning methods. Additionally, the prevailing management policies and practices in China significantly contribute to this issue (Cheng and Hu 2010).

## Discussion

The considerable dioxin emissions reductions in European countries align with restricted waste management policies, incentivized WTE investment, and emission control technologies, such as advanced flue gas cleaning systems (CEWEP 2022). Studies have shown that WTE plants in the European Union have significantly reduced their dioxin emissions due to technological advancements and strict regulations (Hodgkinson et al. 2021; Mathew et al. 2025). The European Environment Agency (2020) documented that landfill emissions decreased by approximately 46% between 1990 and 2018, primarily due to diverting organic waste from landfills. Similar trends have been observed in Japan and Germany, where stringent waste policies and emission controls have led to a drastic reduction in dioxin emissions from WTE facilities (Tun et al. 2020; Yoshida et al. 2009). Ireland’s success, with a 61% reduction over a 6-year period, can be largely attributed to EU-driven waste policies, like the Landfill Directive, which sets ambitious goals for minimizing landfill use and promotes sustainable waste treatment alternatives (European Commission, 2023). This policy framework, along with economic incentives, has facilitated Ireland’s rapid decline in landfill reliance from 60 to 20% over the study period, with WTE filling the gap in managing non-recyclable waste (Department of the Environment, Climate and Communications, 2012; Forfás, 2010; Environmental Protection Agency, 2020).

In contrast, China’s slight increase in dioxin emissions highlights challenges to countries with rapidly growing waste volumes and an existing reliance on landfill infrastructure. Despite a substantial increase in WTE adoption, China’s rapidly increasing MSW generation rate, lower landfill restrictions, and differences in incineration technology have limited the impact of WTE on emission reductions. These disparities underscore the importance of tailored waste management strategies that account for local environmental policies, waste volumes, and technology adoption levels to achieve substantial dioxins reductions (Zhang et al. 2015).

This study demonstrates that adopting WTE technology offers a practical solution for reducing dioxin emissions, especially during the critical early stages of waste management improvements. By transitioning from landfilling to WTE, dioxin emissions can be immediately decreased due to reduced landfill fires and controlled combustion processes in WTE facilities. As presented in the results of this study, European countries serve as successful examples, showing how WTE adoption has led to significant reductions in harmful emissions, aligning with EU policy goals for cleaner and more sustainable waste treatment systems.

However, the financial feasibility of WTE adoption in developing countries remains a significant challenge. The initial investment required for WTE facilities, including incineration plants with advanced flue gas treatment, is considerably higher than traditional landfilling (Khan et al. 2022). While the long-term benefits, such as reduced air pollution, lower healthcare costs from dioxin exposure, and energy recovery outweigh these costs, funding remains a critical barrier (Küfeoğlu 2024). In regions with constrained financial resources, public–private partnerships (PPPs), international funding, and phased implementation strategies could enhance the economic viability of WTE projects (Tahir et al. 2024).

Policy frameworks play a crucial role in enabling WTE adoption. The EU’s Landfill Directive has successfully driven landfill reductions by imposing strict waste diversion targets and promoting alternative waste treatment solutions (European Environment Agency 2020). However, direct replication of this policy approach in developing regions may require adaptation to local economic conditions. Policies such as tax incentives for WTE investments, carbon credit mechanisms, and landfill bans for high-calorific waste could accelerate WTE adoption in countries facing landfill overcapacity. Additionally, aligning waste policies with international climate commitments, such as the Paris Agreement and the Global Methane Pledge, could encourage developing nations to integrate WTE as part of their sustainable waste management strategies (UN, Framework Convention on Climate Change, 2018). Similar landfill-diversion laws outside Europe, Japan’s national surcharge scheme, Singapore’s *Zero-Waste Masterplan* and China’s *Circular-Economy Five-Year Plan*—have each cut the share of municipal solid waste landfilled to ≤ 10% within roughly a decade (MOEJ 2025; NEA 2024; Wei et al. 2022). Comparative reviews show that three design features underlie their success: a steadily escalating landfill fee or cap, earmarking of the revenue for recycling or WTE investment, and compulsory weigh-bridge reporting for enforcement (OECD 2019).

For developing and underdeveloped regions, WTE technology offers a promising approach to addressing the urgent need for waste management improvements while simultaneously reducing public health risks associated with dioxins exposure. As a rapid solution, WTE can provide immediate benefits in reducing toxic emissions compared to traditional landfilling, while recycling and resource recovery systems can be developed as complementary, longer-term solutions. Countries with waste management infrastructures that are adaptable to WTE could effectively use this technology to mitigate dioxin emissions while creating pathways for a more circular waste management system.

For countries with a high dependence on landfilling, policy initiatives aimed at promoting WTE technology and strengthening regulatory frameworks could support emissions reduction efforts (Hsu et al. 2024). Although China has already invested significantly in WTE plants and established emission regulations (including dioxins limits) comparable to EU standards, the country’s rapid economic development has driven substantial increases in waste generation (Lee et al. 2020). Therefore, an increasing approach to WTE investment alongside recycling infrastructure development may be more suitable for China. Alternatively, these recommendations could apply more broadly to developing countries where establishing emissions limits for dioxins from landfill fires, incentivizing cleaner WTE technologies, and gradual landfill phase-out strategies are still needed. Furthermore, international collaboration on technology transfer could enable access to advanced WTE systems and pollution control technologies in these regions.

The success of EU countries in achieving dioxins reductions through the Landfill Directive and emissions standards suggests that adopting similar policy measures in developing regions could promote sustainable waste treatment. For instance, policies that set annual landfill reduction targets or require regular monitoring of dioxin emissions could encourage waste management practices that prioritize WTE and recycling, thereby improving a transition to more sustainable and health-conscious waste management systems (European Commission, 2023).

Public concerns about dioxin emissions from waste incineration partly stem from a lack of data on uncontrolled sources of these emissions. The absence of systematic emission data, particularly for landfill fires, complicates comparisons when transitioning to different waste treatment methods. This data gap has led many citizen activists and organizations to direct their opposition toward waste incineration based on its toxic emissions, potentially overlooking broader implications. Our study seeks to provide a numerical estimate and clarify this relatively understudied issue, contributing to a more informed understanding of dioxin emissions sources.

Although WTE is not the ultimate solution in circular economy models, it serves as an important transitional technology, particularly for non-recyclable and organic waste. By reducing reliance on landfills, WTE contributes to a circular economy by efficiently managing residual waste and decreasing the environmental load of dioxins and greenhouse gases. As circular economy principles gain traction globally, WTE could play a complementary role alongside recycling and resource recovery efforts, filling critical gaps where waste streams are complex and challenging to recycle (CEWEP 2022).

The financial feasibility of WTE technologies is a critical factor influencing their adoption, particularly in developing countries. While WTE requires substantial initial investment, the long-term environmental and economic benefits, such as reduced pollution, lower healthcare costs from dioxins exposure, and energy recovery, make it a viable solution for sustainable waste management. The EU’s transition away from landfilling was driven by strict environmental regulations, economic incentives, and investment in advanced waste treatment infrastructure. While this approach has proven effective, its direct replication in other regions may require adaptation to local economic conditions and policy frameworks. Developing countries may need tailored financial support mechanisms, such as public–private partnerships, international funding, or gradual policy implementation, to facilitate the transition from landfilling to WTE. Understanding these challenges and opportunities is essential for ensuring a successful waste management transformation at a global scale.

## Conclusion

The transition from landfill to WTE in the municipal solid waste management system has significant effects on dioxin emissions, which are harmful pollutants with a strong environmental impact. In many countries, there is still objection of waste incineration partly based on the fear of dioxin emissions. It has been shown in earlier research that landfills are the main source of dioxin emissions in waste management. The idea of this study was to estimate with a rough calculation the magnitude of the dioxin emissions change in such countries which have reduced landfilling rapidly by transitioning to WTE.

Results indicate and validate the effectiveness of WTE technology in minimizing dioxin emissions compared to traditional disposal methods that result in landfill fires. The results show that the reduction of dioxin emissions related to landfilling and WTE in all countries was between 27 and 94% per mass unit of MSW treated by WTE or landfilling. The decrease was primarily attributed to the reduction of landfill fires, which have been identified in the study by Dwyer and Themelis (2015), as one of the major sources of dioxin emissions. The extent of the emission reduction was affected mostly by the relative reduction in landfilling and increase in WTE.

Landfilling is still the main treatment method of MSW globally and especially in the developing countries. Recycling and material recovery are the most sustainable ways to utilize waste, but WTE is needed to complement them because in MSW there are considerable share of such mixed and organic compounds containing waste materials that are technically and economically almost impossible to get recycled. WTE has also been shown in practice to be a rapid possibility to reduce landfilling. Such a way WTE can also effectively reduce methane emissions of landfilling, which are contributing strongly to climate change, and are very important to mitigate during the critical near future decades. This study demonstrates that reduction of landfilling with current WTE technologies has very good potential to reduce dioxin emissions of waste management, and this fact can be used as one justification for WTE investments.

Furthermore, for underdeveloped and developing countries, the results from selected EU countries provide a model for leveraging WTE as a viable waste management solution with immediate environmental and public health benefits. While WTE presents a rapid solution to dioxins pollution, recycling and circular economy practices remain essential long-term strategies for sustainable waste management. By balancing WTE adoption with enhanced recycling efforts, countries can work toward a circular economy while reducing the immediate burden of dioxin emissions.

## Supplementary Information

Below is the link to the electronic supplementary material.

ESM 1 (DOCX 174 KB)

## Data Availability

The datasets generated and analyzed during the current study are not publicly available due to confidentiality agreements, but they are available from the corresponding author upon reasonable request.

## References

[CR1] Ajay SV, Kirankumar PS, Varghese A, Prathish KP (2022) Assessment of dioxin-like POPs emissions and human exposure risk from open burning of municipal solid wastes in streets and dumpyard fire breakouts. Exposure Health 14(3):763–778. 10.1007/s12403-021-00450-4

[CR2] CEWEP – Confederation of European Waste-to-Energy Plants (2023) *Report – Dioxins and Waste-to-Energy: State of the art*. Available at: https://www.cewep.eu/dioxins-wte-state-of-the-art/ (Accessed: June 2025)

[CR3] CEWEP – Confederation of European Waste-to-Energy Plants (2022) *Dioxins and WtE plants: State of the art – European-wide overview of long-term analysis of dioxins in WtE plant surroundings*. Supported by ESWET. Available at: http://eswet.eu/wp-content/uploads/2022/03/CEWEP-Report-Dioxins-and-WtE-plants-State-of-the-Art-1.pdf (Accessed: June 2025)

[CR4] Cheng H, Hu Y (2010) Curbing dioxin emissions from municipal solid waste incineration in China: re-thinking management policies and practices. Environ Pollut 158(9):2809–2814. 10.1016/j.envpol.2010.06.014 20619516 10.1016/j.envpol.2010.06.014

[CR5] Cherubini F, Bargigli S, Ulgiati S (2009) Life cycle assessment of waste management strategies: landfilling, sorting plant and incineration. Energy 34(12):2116–2123. 10.1016/j.energy.2008.08.023 10.1016/j.wasman.2007.11.011 18230413

[CR6] CSIR-National Institute for Interdisciplinary Science & Technology (2021) *Study report on the emission of dioxins and furans during the fire breakout at Brahmapuram Waste Treatment Plant – February 2020*. Thiruvananthapuram: CSIR-NIIST. Available at: https://images.assettype.com/thefourthonline/2023-03/... (Accessed: June 2025)

[CR7] Cudjoe D, Acquah PM (2021) Environmental impact analysis of municipal solid waste incineration in African countries. Chemosphere 265:129186. 10.1016/j.chemosphere.2020.129186 33307505 10.1016/j.chemosphere.2020.129186

[CR8] Cudjoe D, Yuan Q, Han MS (2020) An assessment of the influence of awareness of benefits and perceived difficulties on waste sorting intention in Beijing. J Clean Prod 272:123084. 10.1016/j.jclepro.2020.123084

[CR9] Dąbrowska D, Witkowski AJ, Sołtysiak M (2018) ‘Application of pollution indices for spatiotemporal assessment of negative impact of a municipal landfill on groundwater (Tychy, southern Poland).’ Geol Q 62(3):741–753. 10.7306/gq.1420

[CR10] Dwyer H, Themelis NJ (2015) Inventory of U.S. 2012 dioxin emissions to atmosphere. Waste Manag 46:242–246. 10.1016/j.wasman.2015.08.009 26297638 10.1016/j.wasman.2015.08.009

[CR11] ESWET – European Suppliers of Waste-to-Energy Technology (2022) ‘Dioxins and Waste-to-Energy plants: New report on the state of the art’, *ESWET News*, 28 March. Available at: https://eswet.eu/dioxins-and-waste-to-energy-plants-new-report-on-the-state-of-the-art/ (Accessed: June 2025)

[CR12] Ettala M, Rahkonen P, Rossi E, Mangs J, Keski-Rahkonen O (1996) Landfill fires in Finland. Waste Manag Res 14(4):377–384. 10.1006/wmre.1996.0038

[CR13] European Environment Agency (2020) *The European Environment – State and Outlook 2020: Diversion of waste from landfill*. Available at: https://www.eea.europa.eu/en/analysis/indicators/diversion-of-waste-from-landfill (Accessed: June 2025)

[CR14] Eurostat (2022) *Municipal waste by treatment*. Dataset. Available at: https://ec.europa.eu/eurostat/ (Accessed: June 2025)

[CR15] Falsafi, A.S. (2023) *Speed of transition of municipal solid waste from landfill to waste-to-energy*. Master’s thesis, LUT University. Available at: https://lutpub.lut.fi/handle/10024/165387 (Accessed: June 2025)

[CR16] Forfás (2010) *Waste management in Ireland: benchmarking analysis and policy priorities – Update 2010*. Dublin: Government of Ireland. (Accessed: June 2025)

[CR17] Hodgkinson I, Maletz R, Simon FG, Dornack C (2021) Mini-review of waste-to-energy related air pollution and their limit value regulations in an international comparison. Waste Manag Res 40(7):849–858. 10.1177/0734242x211060607 34823392 10.1177/0734242X211060607

[CR18] Hsu HW, Binyet E, Nugroho RAA, Wang WC, Srinophakun P, Chein RY, Demafelis R, Chiarasumran N, Saputro H, Alhikami AF, Sakulshah N, Laemthong T (2024) Toward sustainability of waste-to-energy: an overview. Energy Convers Manage 321:119063. 10.1016/j.enconman.2024.119063

[CR19] IPCC (2006) *2006 IPCC Guidelines for National Greenhouse Gas Inventories, Vol. 5, Ch. 5: Incineration and Open Burning of Waste*. Available at: https://www.ipcc-nggip.iges.or.jp/public/2006gl/pdf/5_Volume5/V5_5_Ch5_IOB.pdf (Accessed: June 2025)

[CR20] Khan I, Chowdhury S, Techato K (2022) Waste-to-energy in developing countries: opportunities, challenges, and policies. Sustainability 14(7):3740. 10.3390/su14073740

[CR21] Küfeoğlu S (2024) ‘Waste management emissions.’ Energy and environmental economics. Springer, Cham, pp 555–611. 10.1007/978-3-031-70322-5_11

[CR22] Kulkarni PS, Crespo JG, Afonso CAM (2008) Dioxins sources and current remediation technologies: a review. Environ Int 34(1):139–153. 10.1016/j.envint.2007.07.009 17826831 10.1016/j.envint.2007.07.009

[CR23] European Commission (2023) *Landfill waste*. Directorate-General for Environment. Available at: https://environment.ec.europa.eu/topics/waste-and-recycling/landfill-waste_en (Accessed: June 2025)

[CR24] Lee RP, Meyer B, Huang Q, Voss R (2020) Sustainable waste management for zero-waste cities in China: potential, challenges and opportunities. Clean Energy 4(3):169–201. 10.1093/ce/zkaa013

[CR25] Luo H, Cheng Y, He D, Yang E (2019) Review of leaching behavior of municipal solid waste incineration ash. Sci Total Environ 668:90–103. 10.1016/j.scitotenv.2019.03.004 30852230 10.1016/j.scitotenv.2019.03.004

[CR26] Mathew N, Somanathan A, Tirpude A, Pillai AM, Mondal P, Arfin T (2025) Dioxins and their impact: a review of toxicity, persistence, and novel remediation strategies. Anal Methods 17:1698–1748. 10.1039/d4ay01767f 39878532 10.1039/d4ay01767f

[CR27] Ministry of the Environment, Japan (2025) *Municipal solid waste generation and disposal in FY2023*. Press release, 14 March. Tokyo: MOEJ. Available at: https://www.env.go.jp/en/press/press_03835.html (Accessed: June 2025)

[CR28] NASA (n.d.) *Fire information for resource management system (FIRMS)*. Available at: https://firms.modaps.eosdis.nasa.gov/ (Accessed: June 2025)

[CR29] National Bureau of Statistics of China (2020) *China Statistical Yearbook 2020*. Beijing: NBS. Available at: https://www.stats.gov.cn/sj/ndsj/2020/indexeh.htm (Accessed: June 2025)

[CR30] National Environment Agency, Singapore (2024) *Integrated Sustainability Report 2021–2022*. Singapore: NEA. Available at: https://www.nea.gov.sg/docs/default-source/isr/nea-integrated-sustainability-report-2021-2022.pdf (Accessed: June 2025)

[CR31] OECD (2019) *Waste management and the circular economy in selected OECD countries: evidence from environmental performance reviews*. Paris: OECD Publishing. 10.1787/9789264309395-en (Accessed: June 2025)

[CR32] Örebro University (2022) *Global monitoring plan on persistent organic pollutants*. Available at: https://www.oru.se (Accessed: June 2025)

[CR33] Ruokojärvi P, Ettala M, Rahkonen P, Tarhanen J, Ruuskanen J (1995) PCDDs and PCDFs in municipal waste landfill fires. Chemosphere 30(9):1697–1708. 10.1016/0045-6535(95)00055-d

[CR34] Schuhmacher M, Domingo JL (2006) Long-term study of environmental levels of dioxins and furans in the vicinity of a municipal solid waste incinerator. Environ Int 32(3):397–404. 10.1016/j.envint.2005.09.002 16271390 10.1016/j.envint.2005.09.002

[CR35] Šourková M, Adamcová D, Zloch J, Skutnik Z, Vaverková MD (2020) Evaluation of the phytotoxicity of leachate from a municipal solid waste landfill: the case study of Bukov Landfill. Environments 7(12):111. 10.3390/environments7120111

[CR36] Tahir J, Atkinson M, Tian Z, Kassem M, Ahmad R, Martinez P (2024) A critical analysis of public-private partnership models in energy-from-waste projects. Sustainable Futures 8:100240. 10.1016/j.sftr.2024.100240

[CR37] Tan ST, Ho WS, Hashim H, Lee CT, Taib MR, Ho CS (2015) 3E analysis of waste-to-energy strategies for MSW management in Malaysia. Energy Convers Manag 102:111–120. 10.1016/j.enconman.2015.02.010

[CR38] Tun MM, Palacky P, Juchelkova D, Síťař V (2020) Renewable waste-to-energy in Southeast Asia: status, challenges, opportunities, and selection of technologies. Appl Sci 10(20):7373. 10.3390/app10207312

[CR39] U.S. Environmental Protection Agency (2003) *Exposure and human health reassessment of 2,3,7,8-TCDD and related compounds. Part I, Vol. 1, Ch. 6: Combustion sources – minimally controlled and uncontrolled*. Draft, December. Washington, DC: EPA. Available at: https://cfpub.epa.gov/ncea/iris_drafts/dioxin/nas-review/pdfs/part1_vol1/dioxin_pt1_vol1_ch06_dec2003.pdf (Accessed: June 2025)

[CR40] U.S. Environmental Protection Agency (2006) *An inventory of sources and environmental releases of dioxin-like compounds in the United States for the years 1987, 1995, and 2000 (Final, Nov 2006)*. Washington, DC: EPA. Available at: https://cfpub.epa.gov/ncea/risk/recordisplay.cfm?deid=159286 (Accessed: June 2025)

[CR41] U.S. Environmental Protection Agency (2013) *Advancing sustainable materials management: facts and figures 2013*. Washington, DC: EPA. Available at: https://www.epa.gov/sites/default/files/2015-09/documents/2013_advncng_smm_fs.pdf (Accessed: June 2025)

[CR42] UNEP – United Nations Environment Programme (2012) *Toolkit for identification and quantification of releases of dioxins, furans and other unintentional POPs*. Geneva: UNEP. Available at: https://toolkit.pops.int/publish/downloads/unep-pops-toolkit-2012-en.pdf (Accessed: June 2025)

[CR43] Viel J, Floret N, Deconinck E, Focant J, De Pauw E, Cahn J (2011) Increased risk of non-Hodgkin lymphoma and serum organochlorine concentrations among neighbors of a municipal solid waste incinerator. Environ Int 37(2):449–453. 10.1016/j.envint.2010.11.009 21167603 10.1016/j.envint.2010.11.009

[CR44] Wang Y, Yan Y, Chen G, Zuo J, Yan B, Yin P (2017) Effectiveness of waste-to-energy approaches in China from the perspective of greenhouse gas reduction. J Clean Prod 163:99–105. 10.1016/j.jclepro.2015.09.060

[CR45] Wei J, Li H, Liu J, Zhong R (2022) National and provincial dioxin emissions from municipal solid waste incineration in China. Sci Total Environ 851:158128. 10.1016/j.scitotenv.2022.158128 35987242 10.1016/j.scitotenv.2022.158128

[CR46] Weichenthal S, Van Rijswijk D, Kulka R, You H, Van Ryswyk K, Willey J, Dugandzic R, Sutcliffe R, Moulton J, Baike M, White L, Charland J-P, Jessiman B (2015) ‘The impact of a landfill fire on ambient air quality in the North: a case study in Iqaluit. Canada’, Environmental Research 142:46–55. 10.1016/j.envres.2015.06.018 26093783 10.1016/j.envres.2015.06.018

[CR47] World Health Organization (2016) *Dioxins and their effects on human health*. Fact sheet. Geneva: WHO. Available at: https://www.who.int/news-room/fact-sheets/detail/dioxins-and-their-effects-on-human-health (Accessed: June 2025)

[CR48] Yoshida H, Takahashi K, Takeda N, Sakai S-I (2009) Japan’s waste management policies for dioxins and PCBs. J Mater Cycles Waste Manage 11(3):229–243. 10.1007/s10163-008-0235-z

[CR49] Zero Waste Europe (2023) ‘Long-awaited revamp of the Industrial Emissions Directive improves dioxins monitoring in incinerators’, *Press release*. Available at: https://zerowasteeurope.eu/press-release/long-awaited-revamp-of-industrial-emissions-directive-improves-dioxins-monitoring-in-incinerators/ (Accessed: June 2025)

[CR50] Zhang D, Huang G, Xu Y, Gong Q (2015) Waste-to-energy in China: key challenges and opportunities. Energies 8(12):14182–14196. 10.3390/en81212422

[CR51] Zhang M, Buekens A, Li X (2017) Open burning as a source of dioxins. Crit Rev Environ Sci Technol 47(8):543–620. 10.1080/10643389.2017.1320154

