# Supplementary material for: Assessing dioxin emissions change in the transition from landfilling of MSW to waste-to-energy
Source: Environ Sci Pollut Res Int. 2025 Oct 11;33(6):1960–70. doi: 10.1007/s11356-025-37006-x (PMC12960499; doi:10.1007/s11356-025-37006-x)
Supplement: Supplementary file 1 — (DOCX 174 KB) [file 11356_2025_37006_MOESM1_ESM.docx]

**Assessing dioxin emissions change in the transition from landfilling of MSW to waste-to-energy**

**Supplementary Material**

1. Tables:

Tables 1 and 2 present data on the total amount of municipal solid waste (MSW) managed through two primary disposal methods: landfilling and incineration with energy recovery. The values provide insights into national waste management strategies and the shift from landfilling to Waste-to-Energy (WTE) incineration. Understanding the distribution of waste between these two pathways is crucial for assessing changes in dioxin emissions.

Table1. Amount of waste landfill (Eurostat (2022); China Statistical Yearbook (2020))


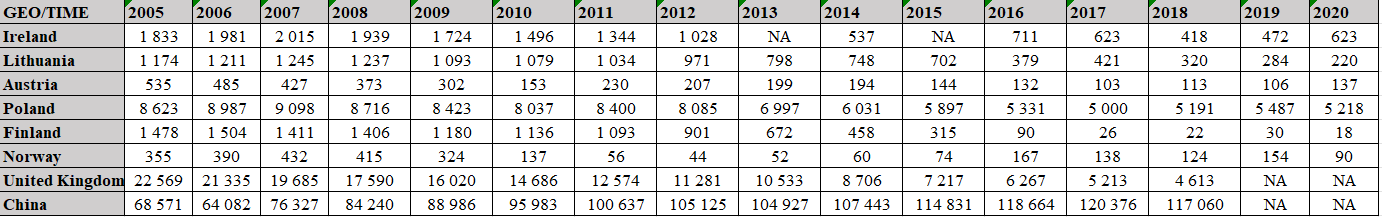


Table 2. Amount of waste incineration with energy recovery (Eurostat (2022); China Statistical Yearbook (2020))


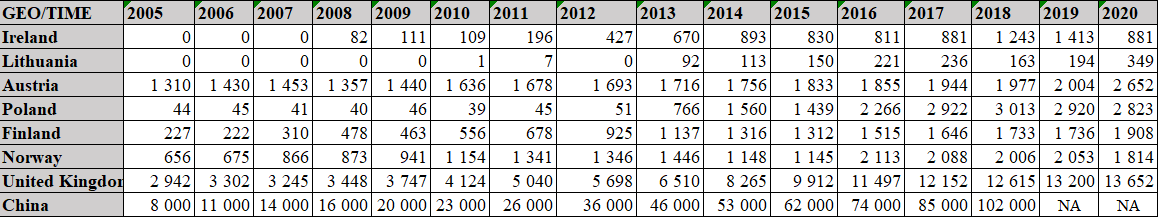


Tables 3 and 4 present the estimated dioxin emissions from two major waste management pathways: **waste-to-energy (WTE) incineration** and **landfill fires**. The values provide a comparison between controlled incineration with energy recovery and uncontrolled landfill fires, offering insights into their respective contributions to dioxin emissions.

Table 3. Dioxin emission from waste-to-energy plants (mg-TEQ)


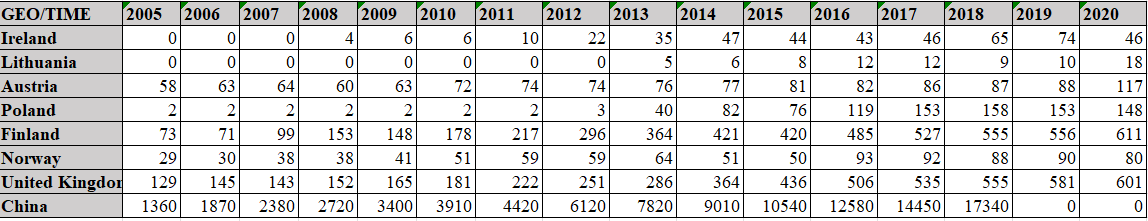


Table 4. Dioxin emission from landfill fire (mg-TEQ)


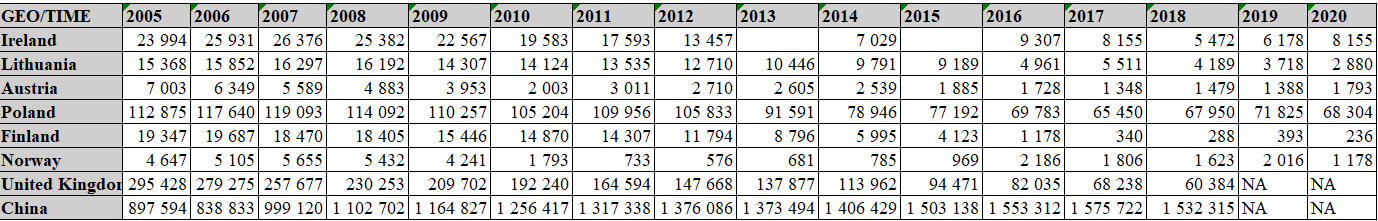


1. Reference

- China Statistical Yearbook 2020. (2023, May 11). https://www.stats.gov.cn/sj/ndsj/2020/indexeh.htm
- European Commission, Landfill waste. (2023, April 12). Environment. https://environment.ec.europa.eu/topics/waste-and-recycling/landfill-waste_en
- Eurostat (2022). (Accessed: 09 June 2023). https://ec.europa.eu/eurostat/databrowser/view/ENV_WASMUN__custom_3212641/default/line?lang=en
